# Estimating the nationwide incidence of coxsackievirus A6-associated hand, foot and mouth disease in China, 2008–2022

**DOI:** 10.1186/s40249-026-01446-5

**Published:** 2026-05-11

**Authors:** Yuanhua Liu, Na Wang, Fengfeng Liu, Ke Li, Michael P. Ward, Nicholas C. Grassly, Wei Tu, Jinming Yu, Wenjin Li, Yu Zhao, Jidan Zhang, Jiawen Dong, Tianren Lu, Mengxin Liu, Zhaorui Chang, Zhijie Zhang

**Affiliations:** 1 Department of Epidemiology and Health Statistics, School of Public Health, Key Laboratory of Public Health Safety, Ministry of Education, Fudan University, Shanghai, 200032 China; 2 Department of Public Health, School of Medicine, Nanjing University of Chinese Medicine, Nanjing, China; 3 Division of Infectious Disease, Key Laboratory of Surveillance and Early-Warning on Infectious Disease, National Key Laboratory of Intelligent Tracking and Forecasting for Infectious Disease, Chinese Center for Disease Control and Prevention, Beijing, 102206 China; 4 Shanghai Institute of Infectious Disease and Biosecurity, Fudan University, Shanghai, China; 5 Sydney School of Veterinary Science, The University of Sydney, Camden, Australia; 6 MRC Centre for Global Infectious Disease Analysis, School of Public Health, Imperial College London, London, UK; 7 Department of Geology and Geography, Georgia Southern University, Statesboro, USA

**Keywords:** Hand, foot and mouth disease, Coxsackievirus A6, Disease burden, Geostatistical model

## Abstract

**Background:**

Due to insufficient routine surveillance, the nationwide disease burden of hand, foot and mouth disease (HFMD) caused by coxsackievirus A6 (CVA6), an emerging serotype, in China remains unclear. This study aimed to estimate the incidence of CVA6-associated HFMD across the Chinese mainland.

**Methods:**

CVA6 positive data from 511 locations across the Chinese mainland during 2008–2022 were integrated from the national pathogen surveillance system and literature, and reported HFMD cases during the same period were obtained from the national infectious disease surveillance system. The predicted positivity rate and incidence of CVA6-associated HFMD in children under five years of age across the Chinese mainland were estimated using a Bayesian geostatistical Gaussian model based on positivity data, reported cases, and environmental, socioeconomic, demographic, and vaccination factors.

**Results:**

The model estimated that the average positivity rate of CVA6 in the Chinese mainland from 2008 to 2022 was 24.1%, with a 95% Bayesian credible interval (BCI) of 11.9–43.3%. The corresponding average annual incidence of CVA6-associated HFMD in children under five years of age was 506 (95% BCI: 272–805) per 100,000. The yearly incidence of CVA6-associated HFMD in children under five years of age peaked in 2018 (873 per 100,000; 95% BCI: 513–1309) before a subsequent decline after 2020. The incidence was highest in South China (1571 per 100,000; 95% BCI: 890–2420) and lowest in Northeast China (208 per 100,000; 95% BCI: 106–340). The estimated CVA6-associated HFMD incidence showed a consistent upward trend across different economic level groups before 2020, and tended to be higher in high-gross domestic product (GDP) per capita regions than in medium- and low-GDP regions.

**Conclusions:**

Model-based estimates indicate a potentially high incidence of CVA6-associated HFMD on the Chinese mainland, particularly in South China, highlighting the need for enhanced surveillance of CVA6 and targeted control efforts in high-incidence regions.

**Graphical Abstract:**

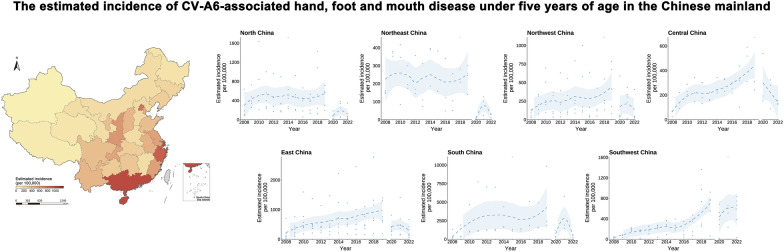

**Supplementary Information:**

The online version contains supplementary material available at 10.1186/s40249-026-01446-5.

## Background

Hand, foot and mouth disease (HFMD) is an infectious disease caused by various enteroviruses, primarily affecting children under five years of age [1]. It is particularly prevalent in subtropical and tropical regions such as China [2], Malaysia [3], Singapore [4] and Vietnam [5]. HFMD caused approximately 96,900 age-weighted disability-adjusted life years, including 96,300 years of life lost, each year across eight high-burden countries in East and Southeast Asia [6]. China is one of the countries with the highest HFMD burden. There were 15,316,710 HFMD cases reported in the Chinese mainland from 2013–2019, and 1,051,262 cases were reported in 2024 [7, 8], which caused a substantial disease burden and represented a considerable public health threat to children’s health. The major serotypes for global circulation of HFMD include enterovirus A71 (EV-A71), coxsackievirus A16 (CVA16), coxsackievirus A6 (CVA6), coxsackievirus A10 (CVA10), and coxsackievirus B3. The composition of these serotypes has undergone a gradual shift, with CVA6 increasingly replacing EV-A71 and CVA16 as the dominant serotype [9]. For instance, CV-A6 was responsible for more than 45% of HFMD cases in both 2015 and 2016 in Shanghai City [10]. During the HFMD outbreak in France in 2021, CV-A6 accounted for 49.5% of HFMD cases [11].

In the Chinese mainland, multiple enterovirus serotypes co-circulate, with EV-A71, CVA16, CVA6, and CVA10 being the major serotypes causing HFMD [12]. Under the current HFMD prevention and control strategies in China, routine surveillance of HFMD associated with EV-A71 and CVA16 facilitated the development of targeted intervention measures and monovalent or multivalent vaccines for these serotypes [13, 14]. Beyond the EV-A71 and CVA16, other serotypes are considered non-routine surveillance serotypes and have not been subject to long-term systematic monitoring. The proportion of these other serotypes gradually increased from 2013 and emerged as the predominant serotypes in 2018, accounting for 70.7% of severe cases [15]. In recent years, other serotypes such as CV-A6 and CV-A10 have gradually emerged as important circulating non-routine surveillance serotypes for HFMD in the Chinese mainland [16, 17].

Since late 2015, the monovalent EV-A71 vaccines was introduced in the Chinese mainland, and the introduction of these vaccines have mostly controlled EV-A71-associated HFMD [18, 19]. However, the vaccine can only prevent EV-A71-associated HFMD and does not provide cross-immunity against other HFMD serotypes [20]. Other enteroviruses have gradually emerged as the leading cause of HFMD [17, 21], and the overall HFMD disease burden remains high [8]. Among these other enteroviruses, CVA6 is gradually emerging as a leading cause of HFMD in China, following its dominance in regions such as Shanghai and Guangxi, where it accounts for over 30% of HFMD cases [10, 22]. The proportion of HFMD outbreaks caused by CV-A6 has also shown an increasing trend nationwide, with approximately 17.05% of outbreaks attributable to CV-A6 following the introduction of the EV-A71 vaccine [12]. However, different HFMD serotypes exhibit different epidemiological patterns [23], and the epidemiological and clinical characteristics of HFMD associated with EV-A71 and CV-A16 do not represent those associated with CVA6. Existing findings from localized areas, such as Shanghai [24], Guangxi [22], and Zhejiang [25], provide some information on the local proportion of CVA6 and showed varying levels of epidemic intensity. In China’s HFMD pathogen surveillance, CVA6 is not routinely identified, with only proximately 13.5% of enterovirus-positive cases being further tested for CVA6 [15]. Therefore, due to inadequate surveillance, the proportion of CV-A6 among all serotypes and the incidence of CVA6-associated HFMD across different areas in the Chinese mainland remains unknown. A comprehensive understanding of the epidemic trends and incidence of CVA6 is therefore vital, as it provides the evidence base to adapt control strategies, strengthen HFMD pathogen surveillance, and prioritize multivalent vaccine development.

Bayesian geostatistical models are a rigorous spatiotemporal statistical approach that leverage limited spatial point observations to infer continuous distributions over an entire region, and they have been widely used to estimate infection risk, case counts, and disease burden of infectious diseases at high spatial resolution [26]. Given that the nationwide incidence of CV-A6-associated HFMD remains unclear, the Bayesian geostatistical model offers a practicable methodological basis for inferring the epidemic situation in non-surveillance areas and predicting the spatial distribution of CV-A6-associated HFMD incidence nationwide.

In this study, we integrated CVA6 positivity rate data from the national HFMD pathogen surveillance system and published literature. Using a Bayesian geostatistical Gaussian model, we predicted the high-resolution spatiotemporal distribution of CVA6 positivity rate. Based on the estimated CVA6 positivity rates and the HFMD reported cases, we estimated the spatiotemporal distribution of incidence of CV-A6-associated HFMD in the Chinese mainland.

## Methods

### Study area

The administrative units of the Chinese mainland include five hierarchical levels. The spatial dimensions, from largest to smallest, are provincial, prefectural, county, township and village levels. These administrative units exist across seven natural geographic regions: (1) Northeast China, (2) East China, (3) North China, (4) Central China, (5) South China, (6) Southwest China, and (7) Northwest China [27].

### Epidemiological data

The annual HFMD reported cases and their age in 31 provincial-level administrative divisions (PLADs) in the Chinese mainland from 2008 to 2022 were obtained from China’s National Notifiable Infectious Diseases Surveillance System, and we included the number of cases in children under five years of age in each PLAD.

We extracted positivity rate data for CVA6 from national pathogen surveillance systems and publicly available literature. The annual serotyping results for CVA6 in available counties from 2016–2018 in Chinese mainland were obtained from the 31 provincial Hand, Foot and Mouth Disease Network Laboratory Pathogen Surveillance System [1, 23]. We estimated the positivity rate using the proportion of positive specimens for CVA6 in the samples tested.

To supplement the national pathogen surveillance data, we conducted a systematic review following the Preferred Reporting Items for Systematic Reviews and Meta-Analyses guidelines [28]. We searched PubMed, Web of Science, ScienceDirect, China National Knowledge Infrastructure (https://www.cnki.net/), and Wanfang Data Knowledge Service Platform (https://www.wanfangdata.com.cn/) from January 1, 2008, to June 30, 2024, without language restrictions, for literature reporting positivity rate data of CVA6 in the Chinese mainland. Detailed methods of the literature search, inclusion/exclusion criteria, study selection, and data extraction are shown in Additional file 1: Text S1–S2.

The positivity rate data for CVA6 at different locations (2008–2022) from the surveillance system and literatures were integrated into the final dataset. If data from a geographic location were duplicated in a given year, we prioritized serotype positivity rate data from surveillance systems or used data from the literatures with the most complete epidemiological information or larger sample sizes.

### Data on covariates

We collected the following covariates from 2008 to 2022 from the Chinese mainland: (1) environmental data including meteorological data, normalized difference vegetation index (NDVI), land cover type, altitude, and slope data; (2) socio-economic data including night light, gross domestic product (GDP) per capita, urbanization rate, and built-up area size; (3) demographic data including population and population density; (4) vaccination rate data. The sources of these covariates and their spatial and temporal resolution are shown in Additional file 1: Table S1. The detailed processing of the covariates is shown in Additional file 1: Text S3. All covariates were transformed into 5 × 5 km^2^ grids across the Chinese mainland, generating a total of 368,756 grids.

### Statistical analysis

#### Variables selection

Because EV-A71 vaccination became available in the Chinese mainland in late 2015, the positivity rate of CVA6 was divided into two survey phases (2008–2015 and 2016–2022) according to the time the data were surveyed. Survey phase, land cover types, and natural geographic regions were included in the model as categorical variables. Spearman correlation analysis was performed for continuous variable, with a coefficient greater than 0.7 indicating high correlation [29], and the highly correlated variables were grouped. Bayesian variable selection was used to determine the optimal covariate set and the optimal functional form of each covariate (linear or categorical [dependent on the quantiles of each variable’s distribution]) (Additional file 1: Text S4) [30]. For each highly correlated variable group, only one explanatory variable was selected, based on the maximum generalized elastic net regression coefficient, to avoid multicollinearity.

#### Bayesian geostatistical model

We constructed a Bayesian geostatistical Gaussian model incorporating spatial random effects to account for spatial processes and included the survey year as a time random effect to address temporal variations, using a first-order autoregressive model. The Bayesian geostatistical Gaussian model, which incorporated the optimal set of covariates and their optimal functional forms, was used to analyze the relationship between the positivity rates of CVA6 and potential environmental, meteorological, vaccination, and socio-economic factors across the Chinese mainland, estimating posterior parameters and their 95% Bayesian credible interval (BCI), with details provided in Additional file 1: Text S5.

A five-fold cross-validation was conducted to evaluate the performance of the final model, using the mean error (ME), mean absolute error (MAE), and the percentage of observed values of the positivity rate included in the 95% BCI of predicted values (Additional file 1: Text S6).

#### High-resolution spatiotemporal distribution of CVA6 positivity rate

The positivity rates for CVA6 were predicted using 5 × 5 km^2^ grids across the Chinese mainland, generating a total of 368,756 grids. The parametric posterior distribution was sampled 300 times to produce 300 sets of predicted values, each set containing 368,756 grid estimates. The median of the predictive values for each grid was used as the estimated positivity rate for that grid, and spatiotemporal distributions of the positivity rates for CVA6 were then mapped.

#### Estimation of the incidence of CVA6-associated HFMD

By integrating the spatiotemporal distribution of predicted CVA6 positivity rates with provincial administrative boundaries, we estimated the positivity rate for CVA6 at the provincial level. The annual number of CVA6-associated HFMD cases among children under five years of age in each PLAD (2008–2022) was estimated by multiplying reported HFMD cases among children under five years of age by the corresponding CVA6 positivity rate. The annual incidence rate was then derived by dividing this case count by the population under five years of age in each PLAD. Based on the estimated annual CVA6-associated HFMD incidence among children under five years of age, we analyzed trends in CVA6-associated HFMD incidence among natural geographic regions and GDP per capita levels from 2008 to 2022 with locally weighted scatterplot smoothing.

The statistical analyses were performed with *terra*, *spikeSlab*, *sf*, *ggplot2* and *INLA* packages in R software (version 4.2.2; R Foundation for Statistical Computing, Vienna, Austria).

## Results

### Characteristics of included positivity rate data for CVA6

**Fig. 1:**
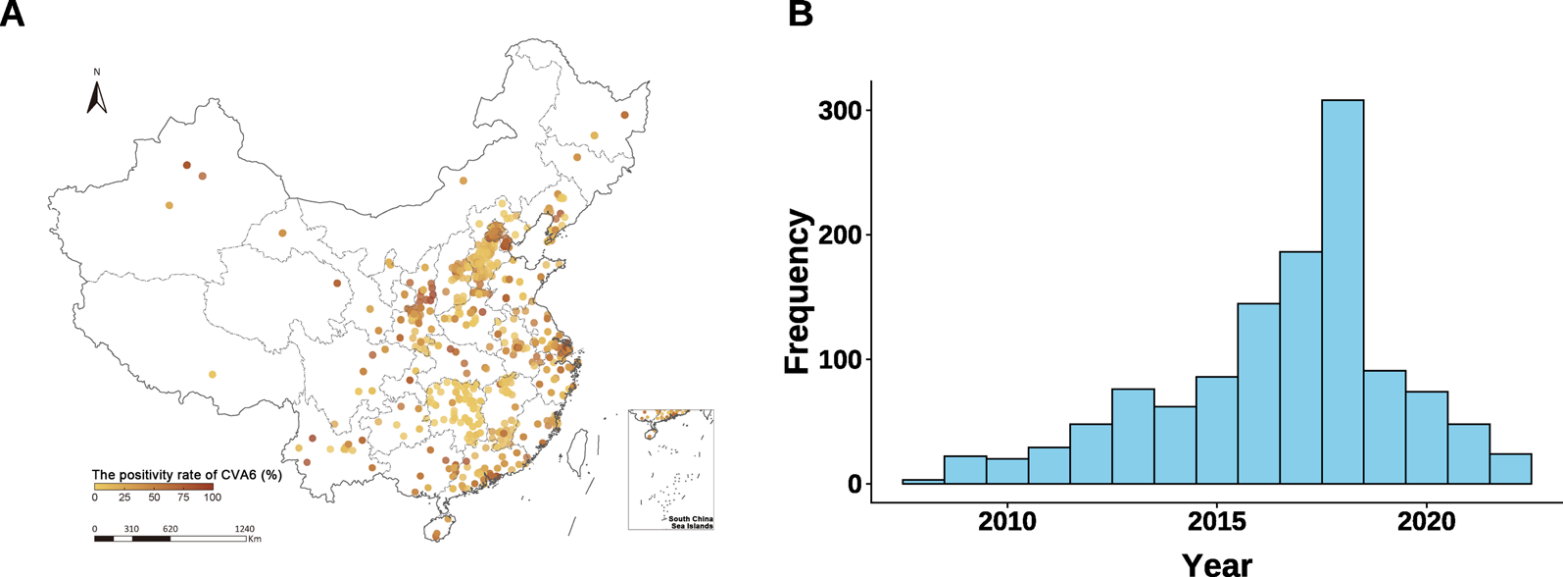
The distribution of raw positivity rate observations, observed locations, and observed years for CVA6. **A** Raw positivity rate observations, and observed locations for CVA6; **B** The yearly distribution of raw positivity rate observations for CVA6. *CVA6* Coxsackievirus A6. Map approval number: GS (2026) 1368

By aggregating data from the Chinese 31 provincial Hand, Foot and Mouth Disease Network Laboratory Pathogen Surveillance System and 476 published surveys of CVA6 (the screening process and summary of the literature included are available in Additional file 1: Text S7–S8, Fig. S1, and Table S2), we obtained 1222 positivity rate observations from 511 unique locations (258 from the pathogen surveillance system and 253 from the published surveys) for CVA6-associated HFMD. These observations were distributed across seven natural regions of the Chinese mainland (Fig. 1A). Among the positivity rate observations from 2008–2022, 28.3% of positivity rate observations were from 2008–2015 (Fig. 1B).

### Results of variable selection

We evaluated 32 variables to construct Bayesian geostatistical Gaussian models for the positivity rates of CVA6 and 12 variables were selected by Bayesian variable selection. The grouping of highly correlated variables, as well as the 12 variables selected and their functional forms included in the final model, are shown in Additional file 1: Tables S3–S4.

### Posterior estimates of the final Bayesian geostatistical model

The final Bayesian geostatistical Gaussian model indicated that GDP per capita, vaccination rate, night light and NDVI were positively associated with the positivity rate of CVA6. Compared to the reference group, the posterior estimates were 0.27 (95% BCI: 0.14–0.41) for the highest GDP per capita group, 0.63 (95% BCI: 0.43–0.83) for the highest vaccination rate group, 0.08 (95% BCI: 0.01–0.15) for the middle NDVI group, and 0.38 (95% BCI: 0.22–0.54) for night light. The parameter estimates for the final Bayesian geostatistical Gaussian model are provided in Additional file 1: Table S4.

### The spatial and temporal distribution of positivity rates for CVA6

**Fig. 2:**
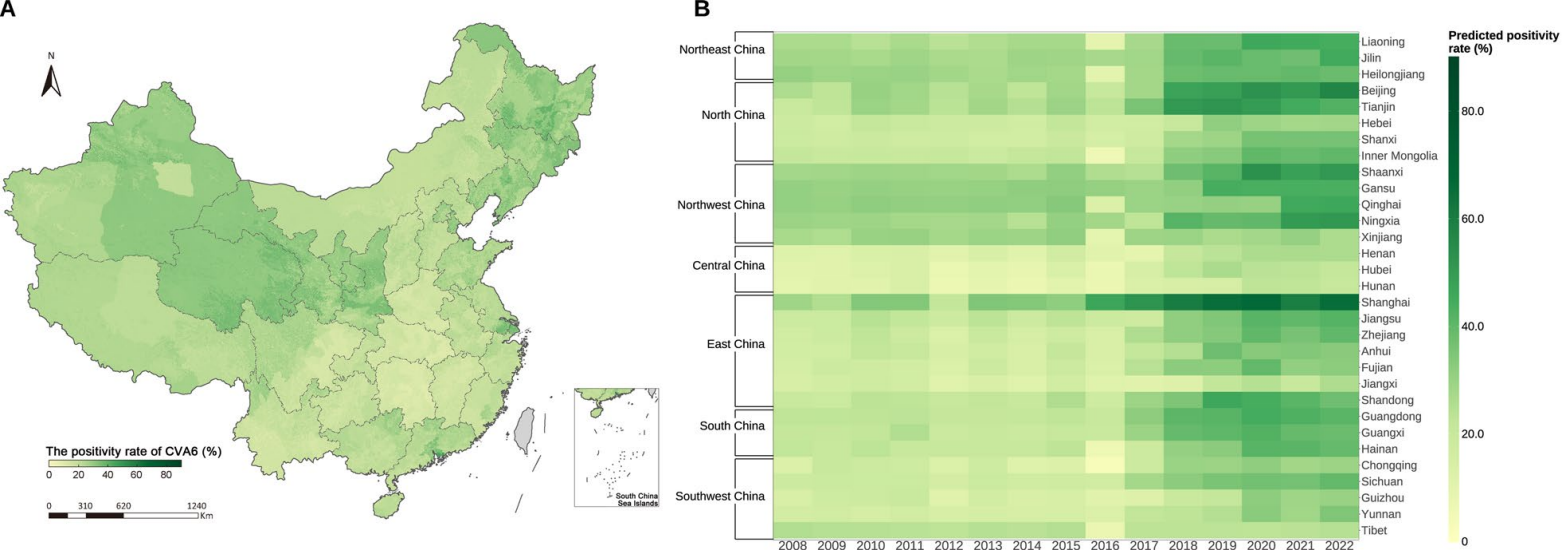
The spatial and temporal distribution of the predicted positivity rates of CVA6 from 2008 to 2022. **A** The spatial distribution of the predicted average annual positivity rates of CVA6 from 2008 to 2022. The grey areas denote that data is not available. The map outline in this figure was obtained from the China Resource and Environment Science and Data Center (http://www.resdc.cn). **B** The temporal distribution of the predicted yearly positivity rates of CVA6 from 2008 to 2022 among 31 PLADs across seven natural geographic regions. *CVA6* Coxsackievirus A6; *HFMD* Hand, foot and mouth disease; *PLAD* Provincial-level administrative division. Map approval number: GS (2026) 1368

The high-resolution spatial distribution of the average annual positivity rates of CVA6 from 2008–2022 is shown in Fig. 2 A. The nationwide average annual positivity rate of CVA6 was 24.1% (95% BCI: 11.9–43.3%) from 2008 to 2022, and the yearly positivity rates exhibited a gradual increasing trend, reaching a peak of 33.9% (95% BCI: 20.1–50.7%) in 2022. The positivity rates for CVA6 gradually increased in the seven natural geographical regions, especially after 2017. Northwest China had the highest CVA6 positivity rate of 30.4% (95% BCI: 16.1–48.5%), followed by Northeast China 28.7% (95% BCI: 15.2%–47.6%), North China 23.8% (95% BCI: 12.7–39.6%), South China 23.0% (95% BCI: 11.9–39.6%), East China 22.4% (95% BCI: 10.9–36.8%), and Southwest China 19.5% (95% BCI: 9.4–34.7%). Central China had the lowest positivity rate for CVA6 of 13.9% (95% BCI: 5.3–24.7%). The positivity rate of CVA6 in these seven regions peaked in 2020–2022 (Fig. 2B). The uncertainty about the predictive positivity rate of CVA6 is shown in Additional file 1: Fig. S2.

### The spatial and temporal distribution of CVA6-associated HFMD incidence among children under five years of age

**Fig. 3:**
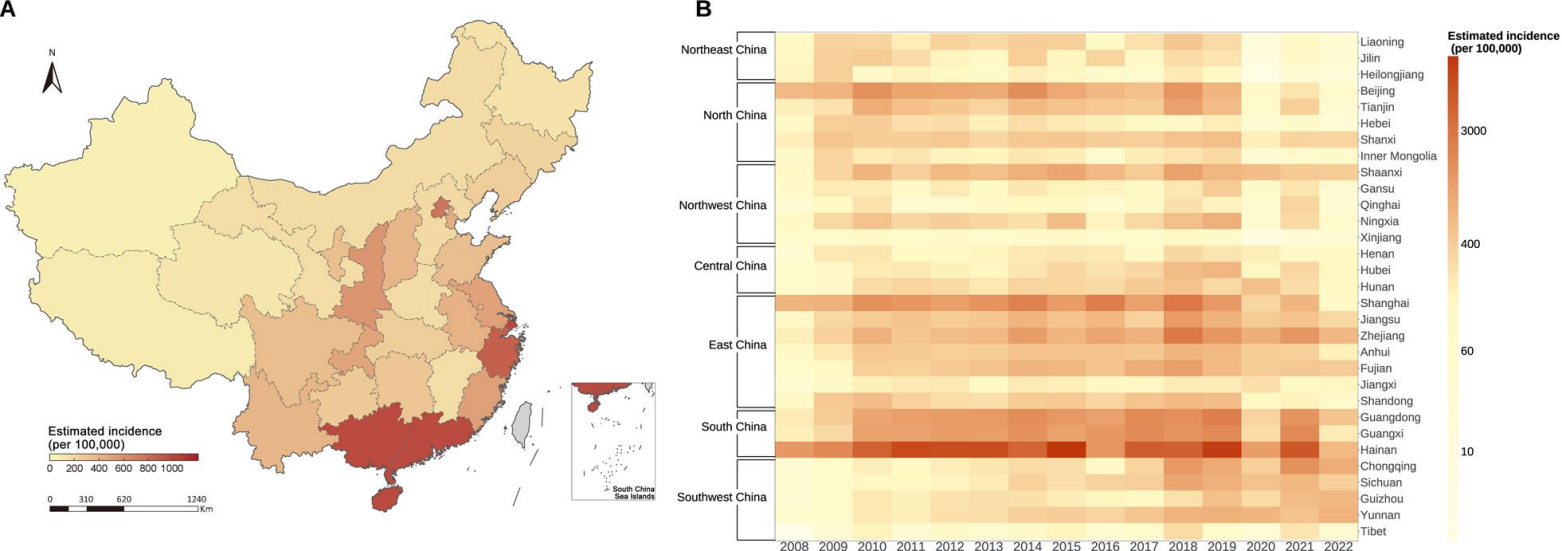
The spatial and temporal distribution of the estimated incidence of CVA6-associated HFMD under five years of age in the Chinese mainland from 2008 to 2022. **A** The spatial distribution of the estimated average annual incidence of CVA6-associated HFMD under five years of age from 2008 to 2022. The grey areas denote that data is not available. The map outline in this figure was obtained from the China Resource and Environment Science and Data Center (http://www.resdc.cn). **B** The temporal distribution of the estimated yearly incidence of CVA6-associated HFMD under five years of age from 2008 to 2022 among 31 PLADs across seven natural geographic regions. *CVA6* Coxsackievirus A6; *HFMD* Hand, foot and mouth disease; *PLAD* Provincial-level administrative division. Map approval number: GS (2026) 1368

**Fig. 4:**
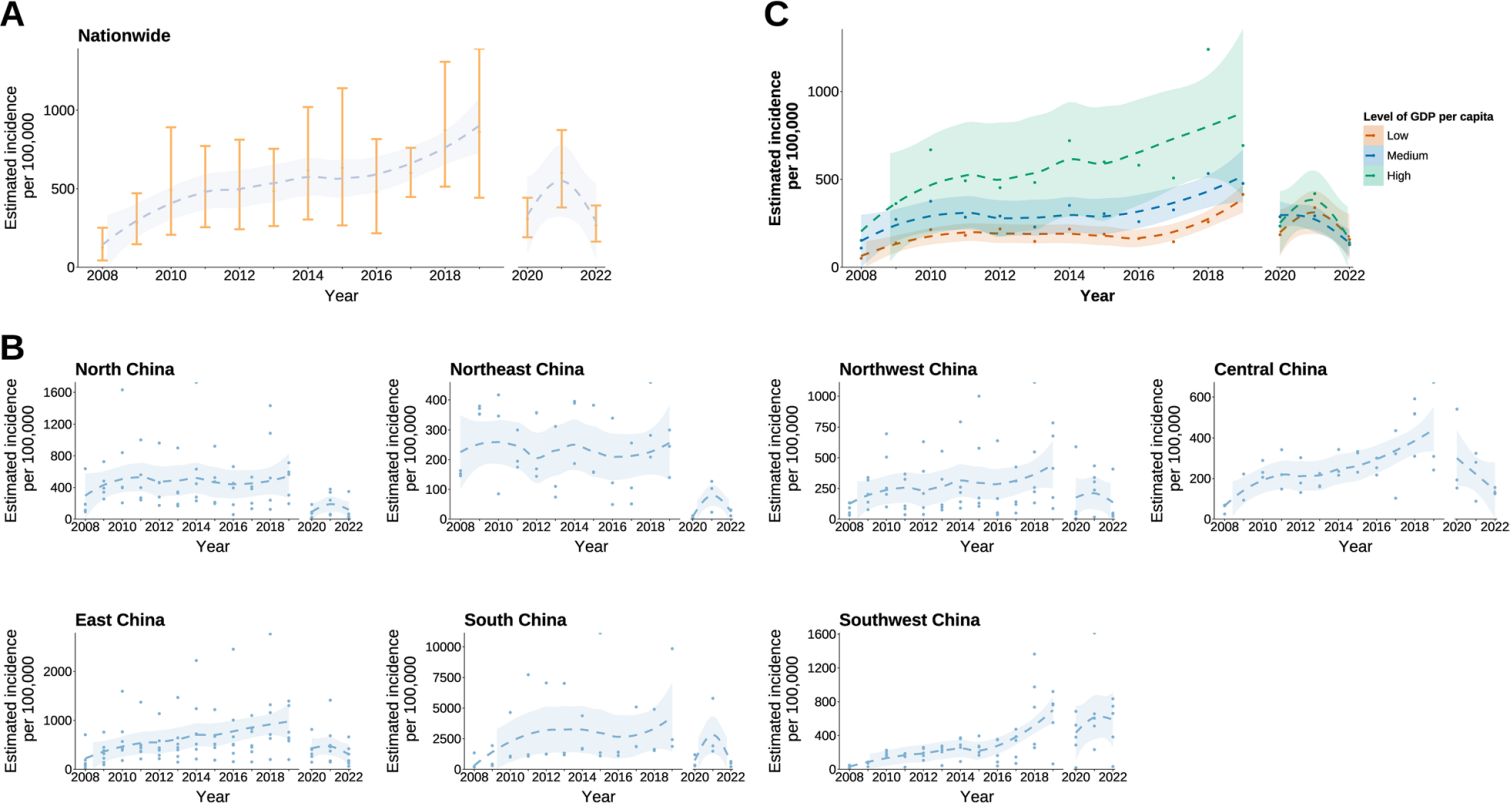
The change in the estimated yearly incidence of CVA6-associated HFMD among seven natural geographic regions and different economic levels. **A** The change of the estimated yearly incidence for CVA6-associated HFMD in children under five years of age nationwide. **B** The change of the estimated yearly incidence for CVA6-associated HFMD in children under five years of age among seven natural geographic regions. **C** The change in the estimated yearly incidence of CVA6-associated HFMD in children under five years of age among different economic levels. The points and error bars indicate the estimated yearly incidence of CVA6-associated HFMD and its 95% BCI for children under five years of age. The dashed line and the shape show the trend and 95% BCI of the estimated yearly incidence for CVA6 fitted using locally estimated scatterplot smoothing. Due to the implementation of nationwide non-pharmaceutical interventions, trends for 2008–2019 and 2020–2022 were fitted separately. *CVA6* Coxsackievirus A6; *HFMD* Hand, foot and mouth disease; *BCI* Bayesian credible interval

The nationwide spatial distribution of the estimated average annual incidence of CVA6-associated HFMD among children under five years of age from 2008 to 2022 is shown in Fig. 3A. The nationwide average annual incidence of CVA6-associated HFMD among children under five years of age was 506 (95% BCI: 272–805) cases per 100,000 individuals from 2008 to 2022, and the yearly incidence gradually increased from 2008 to 2019 and then decreased after 2020, reaching a peak of 873 (95% BCI: 513–1309) in 2018 (Figs. 3B, 4A). South China had the highest CVA6-associated HFMD incidence of 1571 (95% BCI: 890–2420) cases per 100,000 individuals, followed by East China 478 (95% BCI: 260–756), Southwest China 360 (95% BCI: 187–590), North China 293 (95% BCI: 147–482), Northwest China 292 (95% BCI: 159–455), and Central China 292 (95% BCI: 159–455). Northeast China had the lowest CVA6-associated HFMD incidence of 208 (95% BCI: 106–340) cases per 100,000 individuals (Figs. 3B, 4B). The incidence of CVA6-associated HFMD gradually increased from 2008 to 2019 in Northwest China, Central China, East China and South China, then decreased from 2020 to 2022 (Fig. 4B). The incidence of CVA6-associated HFMD among children under five years of age in these four regions peaked in 2018–2019. The incidence of CVA6-associated HFMD in North China and Northeast China remained relatively stable from 2008 to 2019 then gradually decreased, peaking in 2009–2010, while the incidence in Southwest China continued to rise, reaching its peak in 2021 (Fig. 4B).

From 2008 to 2022, the estimated incidence across all economic level groups showed a consistent upward trend, which accelerated after 2016 and peaked in 2019 before declining. High-GDP areas showed the highest incidence, followed by medium-GDP regions, while low-GDP regions had the lowest incidence (Fig. 4C).

### Results of model performance

The Bayesian geostatistical Gaussian model accurately estimated the positivity rate of CVA6 at 77.34% of observations within the 95% BCI. The ME and mean MAE for CVA6 were 1.8% and 12.6%, respectively, indicating that our model may slightly underestimate the positivity rate.

## Discussion

The nationwide incidence of CVA6-associated HFMD among children under five years of age in the Chinese mainland (2008–2022) was estimated for the first time, based on a predicted spatiotemporal distribution of the CVA6 positivity rate derived from a Bayesian geostatistical Gaussian model integrating national pathogen surveillance data and the published literature. These findings are critical for understanding the emerging burden of CVA6-associated HFMD and for guiding targeted surveillance and future vaccine development priorities.

The model estimates indicated that the average positivity rate of CVA6 reached 24.1% during 2008–2022, suggesting that CVA6 has gradually become one of the predominant serotypes of HFMD following EV-A71 and CVA16, consistent with findings from China’s local studies in Jiaxing City of Zhejiang Province, Shanghai Municipality, Xi’an City of Shaanxi Province, and Guizhou Province [31–34]. The model estimated that the average annual incidence of CVA6-associated HFMD among children under five years of age was 506 per 100,000 individuals, showing a gradually increasing trend, particularly after the introduction of the EV-A71 vaccine. These findings suggest that CVA6-associated HFMD has imposed a considerable disease burden among children under five years of age and has gradually become an important public health concern, highlighting the necessity of strengthening surveillance for CVA6. Following the introduction of the EV-A71 vaccine in 2016, a decline in EV-A71-associated HFMD incidence has been observed across the Chinese mainland, particularly in regions with high vaccination coverage, where the GDP per capita is higher, and parents are more willing to vaccinate their children [18, 35–37]. This shift may have created an ecological niche, indirectly facilitating the spread of other serotypes such as CVA6 due to diminished competition from the previously dominant EV-A71 [35]. The high transmissibility of CVA6 relative to other serotypes might make it more likely to spread during multiple serotype co-epidemics, indirectly increasing the proportion of CVA6 [37, 38].

We identified large variations in the incidence of HFMD caused by CVA6 in children under five years of age across regions. The incidence of CVA6-associated HFMD was higher in South China, East China, and Southwest China. The hot and humid climate, high population density, and more advanced economic development of these regions create favorable conditions for the spread of enteroviruses causing HFMD [39, 40]. We found that regions across all economic levels exhibited a persistent and stable stratification in the incidence of CVA6-associated HFMD. High-GDP regions consistently maintained the highest incidence throughout the study period, while low-GDP regions showed relatively lower rates. In economically developed areas, high population density and mobility, which increase contact frequency among susceptible individuals, are associated with a higher incidence. It is similar with previous study [39]. Hence, the incidence of CVA6 is correspondingly high in these regions. It is necessary to develop targeted preventive strategies in these regions. Additionally, regions with low GDP per capita are often more susceptible to variations in healthcare-seeking behavior [41], potentially leading to an underestimation of the incidence of CVA6-associated HFMD. Therefore, strengthening surveillance in low-GDP regions is a crucial foundation for achieving precise nationwide HFMD prevention and facilitates the allocation of public health resources.

The results of the model-estimated spatial distribution of CVA6-associated HFMD incidence provide a scientific basis for optimizing the national HFMD surveillance network, particularly in the placement of sentinel hospitals. The higher incidence of CVA6 in southern China highlights the need to prioritize establishing or strengthening sentinel hospitals at the provincial, municipal, and county levels. This is crucial for ensuring robust pathogen surveillance and effectively tracking CVA6 transmission dynamics. Previous studies indicate that HFMD epidemics exhibit distinct spatiotemporal patterns. Utilizing this characteristic facilitates the rational allocation of sentinel hospitals and enables accurate estimation of HFMD dynamics [42]. We estimated the spatial distribution and dynamics of CVA6 incidence across the Chinese mainland, which facilitates the optimization of the CVA6 sentinel surveillance network and guides the rational allocation of surveillance resources. In addition, to achieve a more comprehensive assessment of the burden of CVA6-associated HFMD, enhanced CVA6 pathogen testing is recommended in resource-limited settings, particularly in regions where CVA6 shows an increasing trend.

Model estimates indicate that, following the widespread EV-A71 vaccination, CVA6 has progressively emerged as one of the major serotypes for HFMD. The current EV-A71 monovalent vaccine offers limited protection and cannot provide cross-serotype immunity. Therefore, the development of multivalent vaccines covering CVA6 may contribute to the prevention and control of HFMD [20]. Multivalent HFMD vaccines that cover CVA6 are currently under development in some countries, such as China [43], the United States [44], and the Republic of Korea [45]. Our estimation of the incidence of CVA6-associated HFMD in children under five suggests the urgent need for such vaccines. Our findings provide a scientific basis for informing future HFMD vaccination strategies and evaluating the public health value of developing multivalent vaccines covering CVA6.

This study has several limitations. First, since survey studies in the literature are mostly summarized annually, this current study lacks a finer temporal scale. However, the year-to-year changes are sufficient to capture the overall trends. Second, while the integration of data from multiple sources presents inherent challenges for assessing uniform diagnostic quality, we addressed this by incorporating the data source as a covariate, which was found to be not statistically significant. Therefore, our analysis relied on the assumption of comparable sensitivity and specificity of diagnostic approaches among various sources. Third, the coverage of the 95% BCIs was generally acceptable but exhibited regional variation. Increasing sample size in future work may help improve the robustness of uncertainty estimates. Fourth, the CVA6 disease burden estimated in this study is based on the number of HFMD surveillance cases, which may underestimate the true disease burden of CVA6. Finally, uncertainty in the estimated incidence was not propagated through a fully joint Bayesian framework, which might underestimate the overall uncertainty of the incidence estimates.

## Conclusions

In this study, a Bayesian geostatistical Gaussian model was employed to predict the incidence of HFMD associated with CVA6 under five years of age from 2008 to 2022. It suggests a potential high incidence of CVA6-associated HFMD in the Chinese mainland, highlighting the need for focused control efforts in high-incidence regions. Furthermore, enhancing the surveillance for CVA6 and developing a multivalent HFMD vaccine including CVA6 might be necessary.

## Supplementary Information

Additional file 1.

## Data Availability

The data and code used and analyzed during the current study available from the corresponding author (epistat@gmail.com) on reasonable request.

## Abbreviations

BCI: Bayesian credible interval
CVA10: Coxsackievirus A10
CVA16: Coxsackievirus A16
CVA6: Coxsackievirus A6
EV-A71: Enterovirus A71
GDP: Gross domestic product
HFMD: Hand, foot and mouth disease
MAE: Mean absolute error
ME: Mean error
NDVI: Normalized difference vegetation index
PLAD: Provincial-level administrative division
