# Supplementary material for: Estimating the nationwide incidence of coxsackievirus A6-associated hand, foot and mouth disease in China, 2008–2022
Source: Infect Dis Poverty. 2026 May 11;15:55. doi: 10.1186/s40249-026-01446-5 (PMC13159216; doi:10.1186/s40249-026-01446-5)
Supplement: Supplementary file 1 — Additional file 1. [file 40249_2026_1446_MOESM1_ESM.pdf]

Additional file 1

Estimating the nationwide incidence of coxsackievirus A6-associated hand, foot and mouth disease in China, 2008–2022

Contents

Supplementary methods **2**

Text S1. The literature search strategy for the survey of Coxsackievirus A6 in the Chinese mainland 2

Text S2. Literature screening and data extraction 4

Table S1. The sources of environmental, socio-economic, demographic and vaccination data 5

Text S3. Processing of the covariates 5

Text S4. Bayesian variable selection 6

Text S5. Bayesian geostatistical model 7

Text S6. Model validation 8

Supplementary results **9**

Text S7. The results of the literature screening 9

Fig. S1. Search and selection process of the locations that reported the positive rates of CVA6 form surveillance system and literature. 10

Text S8. Summary of included literature and locations for CVA6 11

Table S2. Summary of included literature for CVA6 11

Table S3. Results of Bayesian variable selection based on spike-and-slap priors 12

Table S4. Posterior estimates of the final Bayesian geostatistical model for CVA6 positivity rates 13

Fig. S2. The uncertainty of the predicted positive rate of CVA6 in China from 2008 to 2022 14

Reference **15**

Supplementary methods

Text S1. The literature search strategy for the survey of Coxsackievirus A6 in the Chinese mainland

The literature search encompassed 12 common HFMD-associated serotypes to ensure comprehensive coverage. For this study, only literature related to Coxsackievirus A6 (CVA6) were selected for analysis. We did a systematic review following the Preferred Reporting Items for Systematic Reviews and Meta-Analyses guidelines [1], and used the following search terms:

((hand AND foot AND mouth) [Title] OR HFMD [Title] OR CVA2[Title] OR CA2 OR CoxA2[Title] OR Coxsackie 2[Title] OR Coxsackie A2[Title] OR Coxsackievirus A2[Title] OR Coxsackievirus 2[Title] OR CV-A2[Title] OR CVA4[Title] OR CA4[Title] OR CoxA4[Title] OR Coxsackie 4[Title] OR Coxsackie A4[Title] OR Coxsackievirus A4[Title] OR Coxsackievirus 4[Title] OR CV-A4[Title] OR CVA5[Title] OR CA5[Title] OR CoxA5[Title] OR Coxsackie 5[Title] OR Coxsackie A5[Title] OR Coxsackievirus A5[Title] OR Coxsackievirus 5[Title] OR CV-A5[Title] OR CVA6[Title] OR CA6[Title] OR CoxA6[Title] OR Coxsackie 6[Title] OR Coxsackie A6[Title] OR Coxsackievirus A6[Title] OR Coxsackievirus 6[Title] OR CV-A6[Title] OR CVA8[Title] OR CA8[Title] OR CoxA8[Title] OR Coxsackie 8[Title] OR Coxsackie A8[Title] OR Coxsackievirus A8[Title] OR Coxsackievirus 8[Title] OR CV-A8[Title] OR CVA9[Title] OR CA9[Title] OR CoxA9[Title] OR Coxsackie 9[Title] OR Coxsackie A9[Title] OR Coxsackievirus A9[Title] OR Coxsackievirus 9[Title] OR CV-A9[Title] OR CVA10[Title] OR CA10[Title] OR CoxA10[Title] OR Coxsackie 10[Title] OR Coxsackie A10[Title] OR Coxsackievirus A10[Title] OR Coxsackievirus 10[Title] OR CV-A10[Title] OR CVA12[Title] OR CA12[Title] OR CoxA12[Title] OR Coxsackie 12[Title] OR Coxsackie A12[Title] OR Coxsackievirus A12[Title] OR Coxsackievirus 12[Title] OR CV-A12[Title] OR CVB1[Title] OR CB1[Title] OR

CoxB1[Title] OR Coxsackie 1[Title] OR Coxsackie B1[Title] OR Coxsackievirus B1[Title] OR Coxsackievirus 1[Title] OR CV-B1[Title] OR CVB3[Title] OR CB3[Title] OR CoxB3[Title] OR Coxsackie 3[Title] OR Coxsackie B3[Title] OR Coxsackievirus B3[Title] OR Coxsackievirus 3[Title] OR CV-B3[Title] OR CVB4[Title] OR CB4[Title] OR CoxB4[Title] OR Coxsackie B4[Title] OR Coxsackievirus B4[Title] OR CV-B4[Title] OR CVB5[Title] OR CB5[Title] OR CoxB5[Title] OR Coxsackie B5[Title] OR Coxsackievirus B5[Title] OR CV-B5[Title]) AND (((hand AND foot AND mouth) OR HFMD) AND (CVA2 OR CA2 OR CoxA2 OR Coxsackie 2 OR Coxsackie A2 OR Coxsackievirus A2 OR Coxsackievirus 2 OR CV-A2 OR CVA4 OR CA4 OR CoxA4 OR Coxsackie 4 OR Coxsackie A4 OR Coxsackievirus A4 OR Coxsackievirus 4 OR CV-A4 OR CVA5 OR CA5 OR CoxA5 OR Coxsackie 5 OR Coxsackie A5 OR Coxsackievirus A5 OR Coxsackievirus 5 OR CV-A5 OR CVA6 OR CA6 OR CoxA6 OR Coxsackie 6 OR Coxsackie A6 OR Coxsackievirus A6 OR Coxsackievirus 6 OR CV-A6 OR CVA8 OR CA8 OR CoxA8 OR Coxsackie 8 OR Coxsackie A8 OR Coxsackievirus A8 OR Coxsackievirus 8 OR CV-A8 OR CVA9 OR CA9 OR CoxA9 OR Coxsackie 9 OR Coxsackie A9 OR Coxsackievirus A9 OR Coxsackievirus 9 OR CV-A9 OR CVA10 OR CA10 OR CoxA10 OR Coxsackie 10 OR Coxsackie A10 OR Coxsackievirus A10 OR Coxsackievirus 10 OR CV-A10 OR CVA12 OR CA12 OR CoxA12 OR Coxsackie 12 OR Coxsackie A12 OR Coxsackievirus A12 OR Coxsackievirus 12 OR CV-A12 OR CVB1 OR CB1 OR CoxB1 OR Coxsackie 1 OR Coxsackie B1 OR Coxsackievirus B1 OR Coxsackievirus 1 OR CV-B1 OR CVB3 OR CB3 OR CoxB3 OR Coxsackie 3 OR Coxsackie B3 OR Coxsackievirus B3 OR Coxsackievirus 3 OR CV-B3 OR CVB4 OR CB4 OR CoxB4 OR Coxsackie B4 OR Coxsackievirus B4 OR CV-B4 OR CVB5 OR CB5 OR CoxB5 OR Coxsackie B5 OR Coxsackievirus B5 OR CV-B5).

We searched PubMed, Web of Science, ScienceDirect, CNKI, and Wanfang Data Knowledge Service

Platform from Jan 1, 2008, to June 30, 2024, without language restrictions, for surveys that reported HFMD prevalence data caused by CVA6 in the Chinese mainland.

Text S2. Literature screening and data extraction

Inclusion and exclusion criteria

We excluded in-vitro studies, those not reporting on HFMD, non-Chinese mainland studies, case reports, clinical trials, clinical profiles, case-control studies, purely modeling studies, studies that did not differentiate serotypes or only include EV-A71 or CVA16, reviews, editorials, corrections, studies with unavailable full texts, studies that did not report positivity rates, or studies from which positivity rates could not be calculated, studies without location or time information, detection technology studies, studies on vaccines, prevention strategies, economic evaluations, and specific populations (such as cases with neurological symptoms or severe cases).

Screening Process

Initially, we screened titles and abstracts to identify potentially relevant studies and exclude irrelevant literature. We randomly selected 30% of this irrelevant literature to check for misclassification. If misclassified literature existed, we re-screened all the literature. The full text of potentially relevant literature was screened to determine the final studies to be included.

Data extraction process

We extracted epidemiological data for CVA6 including the survey time, survey locations (name, administrative unit), the number of samples tested, the number of positive samples and the positive rate (if the literature reported it), the type of tested samples, the detection method, the survey type, and the references (authors, journals, and date of literature). The geographical coordinates of the survey locations were obtained from Baidu Maps (https://map.baidu.com/). We included studies with sample sizes greater

than ten.

Table S1. The sources of environmental, socio-economic, demographic and vaccination data

Source	Variable	Time	Time resolution	Spatial resolution
National Meteorological Information Centre of China (https://data.cma.cn/)	Average temperature (°C)	2008–2022	Daily	Monitoring station
	Average humidity (%)	2008–2022	Daily	Monitoring station
	Average wind speed (m/s)	2008–2022	Daily	Monitoring station
	Sunshine hours (h)	2008–2022	Daily	Monitoring station
	Precipitation（mm）	2008–2022	Daily	Monitoring station
National Aeronautics and Space Administration of US (https://www.earthdata.nasa.gov/learn/get-started)	Normalized difference vegetation index	2008–2022	Monthly	1×1 km
Zenodo database (https://zenodo.org/records/8176941)	Surface cover type	2008–2022	Yearly	1×1 km
Worldpop database (https://www.worldpop.org/)	Population density (per km^2)	2008–2022	Yearly	1×1 km
National Earth System Science Data Centre of China (http://www.geodata.cn)	Night light (nW/cm2/sr)	2008–2022	Yearly	1×1 km
Resource and Environmental Sciences and Data Centre, Chinese Academy of Sciences (https://www.resdc.cn/)	Altitude (m)	2005	Yearly	1×1 km
	Slope (°)	2005	Yearly	1×1 km
Chinese Statistical Yearbook（https://www.stats.gov.cn/sj/ndsj/）	GDP per capita	2008–2022	Yearly	Prefecture
	Population	2008–2022	Yearly	Prefecture
	Urbanization rate (%)	2008–2022	Yearly	Prefecture
	Built-up area（km^2）	2008–2022	Yearly	Prefecture
Chinese Centre for Disease Control and Prevention	Vaccination coverage	2016–2022	Yearly	Province

Text S3. Processing of the covariates

We processed the collected covariates as follows: (1) The daily meteorological data in the Chinese mainland were obtained from 602 meteorological stations nationwide using kriging interpolation. The annual and HFMD epidemic seasons (April–September)[2] maximum, minimum, and average temperature, humidity, wind speed, accumulated precipitation, and accumulated sunshine hours were aggregated from daily meteorological data; (2) The normalized difference vegetation index (NDVI) among the Chinese mainland was aggregated as the annual mean; (3) Land cover types were aggregated using plurality and classified into eight categories: cropland, forest, scrub, grassland, water and wetlands, snow, wasteland, and impervious surfaces; (4) The percentage of impervious area for each prefecture

was calculated by dividing the total number of impervious surface grids by the total number of grids in the prefecture. (5) The population density of each prefecture was calculated by averaging the population density values for all grids within the prefecture.

All covariates are transformed to 5×5 km^2 grids across the Chinese mainland, generating a total of 368,756 grids.

Text S4. Bayesian variable selection

We conducted spike-and-slab prior Bayesian variable selection to determine the optimal set of covariates. In order to select the best predictor variables, the prior of the static regression component can be set to a spike-and-slab prior, as shown in the following equation:

$$p(\beta) = \pi \cdot p(\beta \mid \beta_0, \sigma^2_{\text{spike}}) + (1 - \pi) \cdot p(\beta \mid \sigma^2_{\text{slab}})$$

The prior of the regression coefficient β mixes two normal distributions, *spike* component with smaller variance and *slab* component with larger variance, and the mixing parameter π controls the weight between these two components. A smaller variance σ^2_{spike} when π is close to 1, the posterior estimate of β shrinks towards 0, implying that the predictor variable is not included in the final model. Conversely, a larger variance σ^2_{slab} when π is close to 0, allows for a non-zero posterior estimate of β, indicating that the predictor variable is included in the final model[3].

Variables were selected if the slab component dominated the posterior distribution, indicated by generalized elastic net (gnet) regression coefficient, and the detailed selection process has been reported in a previous study[4]. The importance of different predictor variables can be compared based on the gnet regression coefficient, the coefficient is greater, the more conducive the predictor variable is to improve the explanation of a model[5]. Bayesian variable selection was used to determine the optimal covariate set and the optimal functional form of each covariate (linear or categorical (dependent on the

quantiles of each variable's distribution)) identified for CVA6 positive rates. For each highly correlated variables group, only an explanatory variable was selected, based on the maximum gnet regression coefficient, to avoid multicollinearity.

Text S5. Bayesian geostatistical model

We constructed Bayesian geostatistical Gaussion models, incorporated spatial random effects to implement spatial processes, and included the year of the survey as a time effect term responding to temporal variations, using the first-order autoregressive model. Bayesian Gaussian geostatistical models were used to analyze the relationship between CVA6 positive rate and potential environmental, meteorological, and socio-economic factors in the Chinese mainland, and to estimate the posterior parameters. Box-Cox transform is applied to CVA6 positive rate, and the positive rate of each serotype Y_{it} follows the Gaussian distribution $Y_{it} \sim Normal(\mu_{it}, \sigma_{it}^2)$ after the transform, the relation between CVA6 positive rate and the potential environmental, climatic, and socio-economic factors are:

$$Y_{it} = \beta_0 + \sum_{j=1}^{n} \beta_j X_{itj} + u_{it}$$

where i is the location (i=1, ..., N), t is the time (t=1, ..., T), β_0 is the intercept, β_j is the regression coefficient of the j-th covariate X_{itj}, and u_{it} is is the latent spatio-temporal process, varying with time:

$$u_{it} = \rho u_{it-1} + \varepsilon_{it}$$

where ρ is the transition parameter with $|\rho| < 1$ in case of stationarity and the instantaneous spatial correlation ε_{it}, independent from u_{it-1}, is distributed according to a multivariate Gaussian distribution $MVN(0, \sum_t)$ with covariance matrix from the Matérn covariance function:

$$\Sigma_{tpq} = Cov(\varepsilon_p, \varepsilon_q) = \frac{\sigma^2}{2^{v-1}\Gamma(v)} (kd_{pq})^v K_v(kd_{pq})$$

d_{pq} is the Euclidean distance between locations p and q, σ^2 is the marginal variance, k is the scale parameter, and K_v is Type II modified Bessel function, v is the smoothing parameter which is usually

fixed at 1 due poor identifiability, Γ is the gamma function. The spatial range is defined as:

$$r = \sqrt{\frac{8v}{k}}$$

with r corresponding to the distance above which spatial dependencies become negligible[6].

Text S6. Model validation

To validate the ultimate model, we conducted 5-fold cross-validation to evaluate its performance. All observations (*N*) were randomly divided into two datasets, with 80% used as the training set and 20% used as the validation set. The training set was used to fit the model and the validation set was used to validate the model, we calculate the following indices to evaluate the model performance: mean error $\left(ME = \frac{1}{n}\sum_{i=1}(\pi_i - \hat{\pi_i})\right)$, mean absolute error ($MAE = \frac{1}{n}\sum_{i=1}|\pi_i - \hat{\pi_i}|$), and the percentage of observative values of the positive rate of each serotype included in the 95% Bayesian Credible Interval (BCI) of the predicted values, where n is the number of validation locations, π_i is the observed value, and $\hat{\pi_i}$ is the predicted value of validation location i.

Supplementary results

Text S7. The results of the literature screening

A total of 10,049 literatures were retrieved and screened for positive rate data for CVA6 according to the inclusion criteria (Fig. S1). The summary of the CVA6-associated HFMD literature included is shown in Text S8 and Table S2. We finally included 476 published surveys of CVA6.

Fig. S1. Search and selection process of the locations that reported the positive rates of CVA6 form surveillance system and literature.

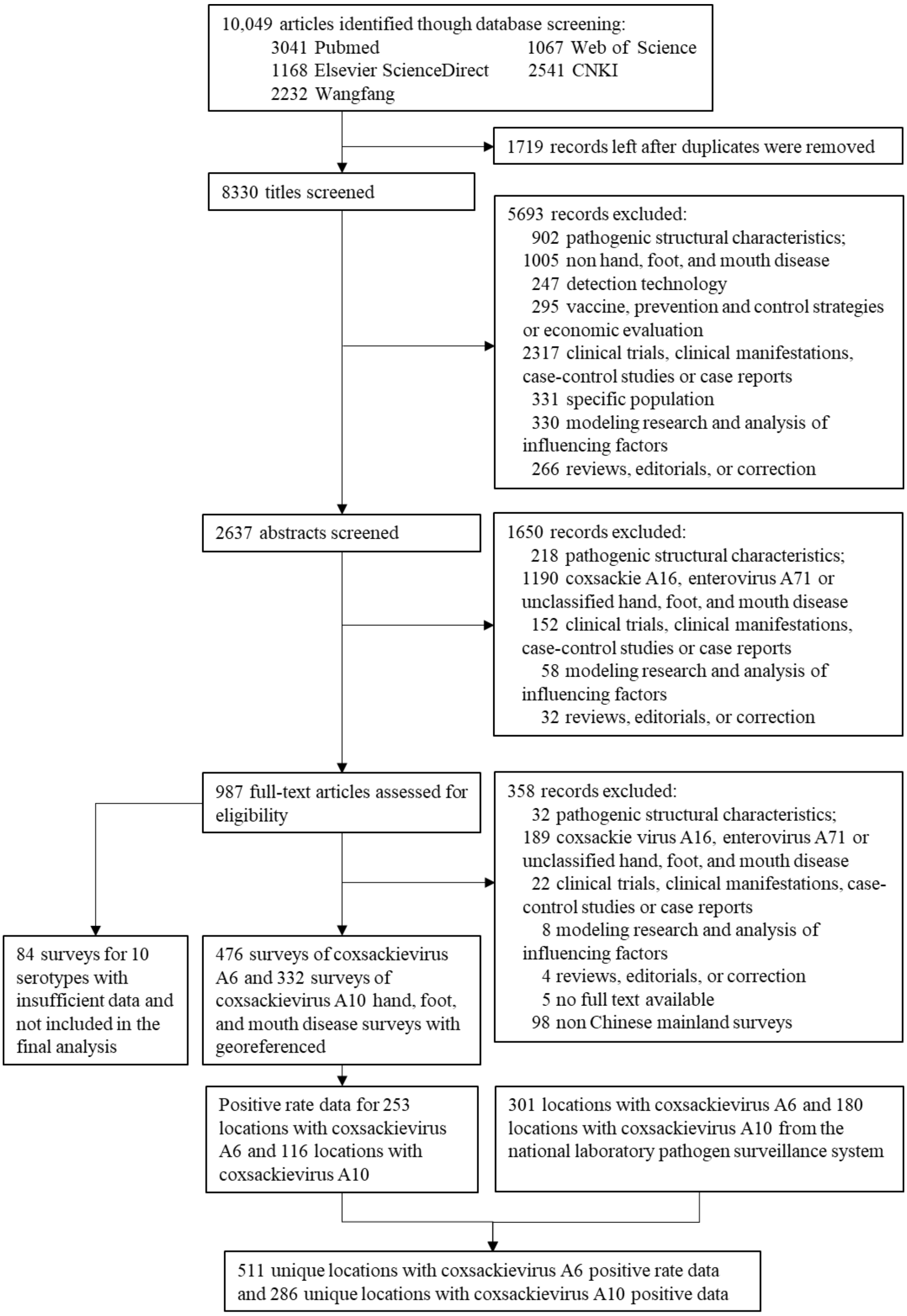

Text S8. Summary of included literature and locations for CVA6

Summary of included literature for CVA6

The CVA6-associated HFMD surveys included were identified using feces (66, 13.9%), anal swabs (17, 3.6%), pharyngeal swabs (128, 26.9%), other (7, 1.5%), and more than two (218, 45.8%) types of samples, and 40 (8.3%) surveys were not described (Table S2).

Summary of included locations for CVA6

From the Provincial Hand, Foot, and Mouth Disease Network Laboratory Pathogen Surveillance System, 301 CVA6 locations were included from 2016–2018. Among the 253 locations included in the CVA6 literature, 96 were surveyed in 2015 and earlier (Phase 1), 57 in 2016 and later (Phase 2), and 100 were surveyed across both stages. After excluding duplicate locations, the study included 511 CVA6 unique locations (253 from the literature and 258 from the pathogen surveillance system) (Table S2).

Table S2. Summary of included literature for CVA6

	**Pre-vaccination (Phase 1, ≤2015)**	**Post-vaccination (Phase 1, ≥2016)**	**Included two phases**	**Total**
Number of literatures	179	206	91	476
Number of locations	96	57	100	253
Sample type				
Feces	31	27	8	66
Rectal swab	6	10	1	17
Throat swab	48	51	29	128
Other	1	6	0	7
≥Two sample types	86	93	39	218
Unspecified	7	19	14	40
Type of survey				
CDC-based surveillance*	140	153	76	369
Hospital-based surveillance	39	52	16	107

*Centers for Disease Control and Prevention

Table S3. Results of Bayesian variable selection based on spike-and-slap priors

Variables	Select
Highly correlated variables	
Group 1	
Annual average temperature	
Annual maximum temperature	
Annual minimum temperature	
Average temperature of epidemic season	
Maximum temperature of epidemic season	
Minimum temperature of epidemic season	√
Group 2	
Annual average humidity	
Annual maximum humidity	
Annual minimum humidity	
Average humidity of epidemic season	
Maximum humidity of epidemic season	
Minimum humidity of epidemic season	
Annual Cumulative annual rainfall	
Cumulative rainfall of the epidemic season	√
Group 3	
Annual average wind speed	√
Average wind speed of epidemic season	
Group 4	
Annual sunshine hours	√
Sunshine hours of the epidemic season	
Group 5	
Population	√
Population under five years of age	
Group 6	
Population density	
Percentage of impervious area	
Night light	√
Group 7	
GDP per capita	√
Urbanization rate	
Built-up area size	
Non-highly correlated variables	
NDVI	√
Vaccination coverage	√
Altitude	
Slope	√
Surface cover type	√
Natural geographic region	√

Table S4. Posterior estimates of the final Bayesian geostatistical model for CVA6 positivity rates

Variables	Median (95% BCI[a])	Variables	Median (95% BCI[a])
Minimum temperature of epidemic season (°C)		Vaccination coverage (%)	
<12.4	Ref	<0.01	Ref
12.4–15.4	-0.48 (-31.51 to 30.54)	0.01–3.3	0.31 (0.13, 0.50)*
>15.4	-0.47 (-31.51 to 30.54)	3.4–10.9	0.52 (0.33, 0.71)*
Annual average wind speed (m/s)		>10.9	0.63 (0.43, 0.83)*
<1.9	Ref	Survey phase	
1.9–2.3	0.01 (-0.06 to 0.09)	2008–2015	Ref
>2.3	-0.04 (-0.14 to 0.05)	2016–2022	-0.36 (-0.55 to 0.17)
Annual sunshine hours (1000 h)	0.13 (-0.03 to 0.27)	Night light (nW/cm^2/sr)	0.38 (0.22, 0.54)*
Cumulative precipitation of the epidemic season (mm)		Slope	
<515.7	Ref	<0.20	Ref
515.7–901.0	-0.03 (-0.10 to 0.04)	0.20–0.90	0.13 (-0.03 to 0.29)
>901.0	-0.07 (-0.17 to 0.03)	0.91–2.27	0.01 (-0.17 to 0.17)
Population/10,000		>2.27	0.02 (-0.16 to 0.21)
<487.33	Ref	NDVI	
487.33–930.64	-0.07 (-0.17 to 0.02)	<0.60	Ref
GDP per capita (CNY)		0.60–0.76	0.08 (0.01, 0.15)*
<42,544	Ref	>0.76	0.02 (-0.07 to 0.11)
42,544–66,878	0.12 (0.04, 0.20)*	Natural geographic region	
66,879–105,387	0.13 (0.04, 0.24)*	Central China	Ref
>105,387	0.27 (0.14, 0.41)*	Eastern China	0.13 (-0.11 to 0.36)
Surface cover type		Northern China	-0.03 (-0.31 to 0.25)
water and wetlands	Ref	Northeastern China	0.32 (-0.05 to 0.67)
forest	-0.07 (-0.22 to 0.08)	Northwestern China	0.23 (-0.04 to 0.50)
scrub	0.08 (-0.08 to 0.24)	South China	0.23 (-0.06 to 0.50)
grassland	-0.17 (-0.36 to 0.04)	Southwest China	0.17 (-0.10 to 0.44)
cropland	0.01 (-0.18 to 0.20)	Spatial range (km)	157.95 (99.17, 230.24)
snow	0.16 (-0.07 to 0.39)	Spatial variance	4.41 (2.57, 6.83)
wasteland	0.03 (-0.16 to 0.21)	Non-spatial variance	0.13 (0.10, 0.17)
impervious surfaces	0.01 (-0.16 to 0.16)		

*Statistically significant.

95% BCI: 95% Bayesian confidence interval. Ref: reference group.

[a]Parameter estimation based on Box-Cox transformed positivity rates for CVA6.

Fig. S2. The uncertainty of the predicted positive rate of CVA6 in China from 2008 to 2022

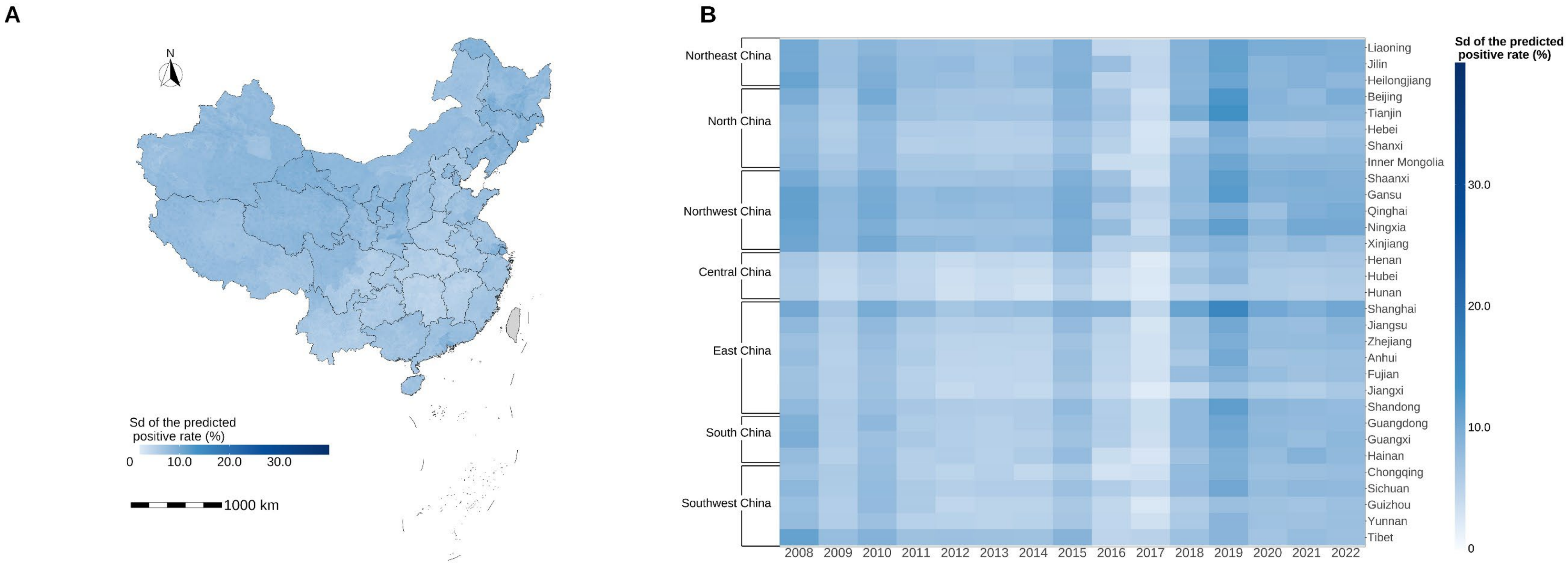

A. The spatial distribution of standard deviation of the predicted average annual positivity rates of CVA6 from 2008 to 2022. The grey areas denote that data is not available.

B. The temporal distribution of standard deviation of the predicted yearly positivity rates of CVA6 from 2008 to 2022 among 31 PLADs across seven natural geographic regions.

Reference

1. Moher D, Liberati A, Tetzlaff J, Altman DG, Group P. Preferred reporting items for systematic reviews and meta-analyses: the PRISMA statement. Ann Intern Med. 2009;151(4):264–9, W64.

2. Xing W, Liao Q, Viboud C, Zhang J, Sun J, Wu JT et al. Hand, foot, and mouth disease in China, 2008-12: an epidemiological study. Lancet Infect Dis. 2014;14(4):308–18.

3. Scheipl F, Fahrmeir L, Kneib T. Spike-and-Slab Priors for Function Selection in Structured Additive Regression Models. Journal of the American Statistical Association. 2012;107(500):1518–32.

4. Ishwaran H, Kogalur UB, Rao JS. spikeslab: Prediction and Variable Selection Using Spike and Slab Regression. R J. 2010;2(2):68–73.

5. Ishwaran H, Rao JS. Spike and slab variable selection: Frequentist and Bayesian strategies. Ann Stat. 2005;33(2):730–73.

6. Luo C, Wang Y, Su Q, Zhu J, Tang S, Bergquist R et al. Mapping schistosomiasis risk in Southeast Asia: a systematic review and geospatial analysis. Int J Epidemiol. 2023;52(4):1137–49.
